# Failure of Guided Endodontic Access in a Severely Obliterated Root Canal: A Case Report of Drill Deviation

**DOI:** 10.1155/crid/5560110

**Published:** 2026-09-29

**Authors:** Stephanie Isabel Diaz Zamalloa, Iandara Lima Scardini, Shirley Maklane Gomes Soares, Daniel de Almeida Decurcio, Marcelo dos Santos, Celso Luiz Caldeira

**Affiliations:** ^1^ Department of Operative Dentistry, School of Dentistry, University of São Paulo, São Paulo, Brazil, usp.br; ^2^ Department of Endodontics, Federal University of Goiás, Goiânia, Goias, Brazil, ufg.br

**Keywords:** computer-assisted, cone-beam computed tomography, dental pulp calcification, digital planning, endodontics, printing, surgery, three-dimensional

## Abstract

Pulp canal obliteration (PCO) increases the risk of procedural errors during canal localization. Guided endodontics improves access accuracy, but its success depends on precise guide design, proper drill–sleeve alignment, and material stability. When these factors are compromised, deviations and unsuccessful canal location may occur. The present clinical case reports the management of a severely obliterated root canal in a maxillary lateral incisor in which static‐guided endodontic failed to achieve canal localization, emphasizing the technical limitations and the successful conventional approach. A 69‐year‐old female patient presented with a maxillary lateral incisor exhibiting severe PCO. Cone‐beam computed tomography (CBCT) confirmed obliteration of the cervical and middle thirds of the root canal. Despite careful planning and the use of a static endodontic guide, canal localization failed after repeated drill deviations toward the palatal aspect. Investigation revealed palatal wear of the resin template adjacent to the metallic sleeve, and the mismatch between the drill′s active length and the guide sleeve′s dimensions may have contributed to the observed deviation. The canal was successfully localized under operating‐microscope magnification using ultrasonic tips, guided by anatomical information obtained from postoperative CBCT imaging. Cleaning, shaping, and obturation were completed without complications. After 3 years, the patient remains asymptomatic, with radiographic signs of satisfactory healing. This case reinforces that guided endodontics—although valuable—requires meticulous planning, precise drill‐sleeve compatibility, and strict guide validation. Clinicians must remain proficient in conventional techniques for cases in which guided access fails.

## 1. Introduction

Pulp canal obliteration (PCO) is a common sequela of dental trauma, particularly after luxation injuries [1, 2]. However, it may also arise from nontraumatic factors, such as age‐related physiological changes, where progressive dentin deposition—often observed in elderly patients due to lifelong secondary dentin formation—leads to canal obliteration [1–8]. Although several terms have historically been used to describe PCO, this condition is characterized radiographically by progressive narrowing of the pulp canal space and should be distinguished from other patterns of pulpal mineralization, such as discrete pulp stones and diffuse pulp mineralizations [9, 10]. In the present report, PCO is used as the principal term to describe this canal‐narrowing process.

Endodontic intervention may be indicated when PCO is associated with irreversible pulpitis or apical pathology [2, 4, 11–14]. However, excessive dentin deposition can hinder root canal localization, impacting the root canal treatment outcome [3, 5, 12].

Radiographically, PCO may present as partial or total obliteration, whereas recent classifications also consider the location and caliber of the remaining canal lumen to characterize the extent of canal narrowing [15, 16]. This narrowing can pose significant clinical challenges. In such scenarios, a conservative approach using static‐guided endodontics is often preferred [8, 17], as it minimizes unnecessary tooth structure removal [11, 18–20], may reduce tooth fracture risk [21], and may mitigate procedural errors such as root perforations, instrument fractures, ledge formation, and failed canal negotiations [5, 13, 20, 22].

The guided approach requires high‐resolution cone‐beam computed tomography (CBCT) with a minimal field of view to visualize obliterated canals, along with a digital surface scan (acquired either through an intraoral scanner or by digitizing a stone model). Specialized planning software merges CBCT images (DICOM file) and surface scan data (STL files), enabling accurate virtual positioning of the access drill toward the visible portion of the canal. A customized virtual guide with a sleeve is subsequently designed and manufactured using either CAD/CAM milling or three‐dimensional (3D) printing technologies [2, 4, 6, 12, 13, 17, 23].

Despite the reported efficacy of static‐guided endodontics [13, 17, 22], the accuracy of this approach depends on multiple factors throughout the digital and clinical workflow, including guide adaptation, the mechanical relationship between the drill and the guiding sleeve, and clinician experience during planning and clinical execution [12, 24, 25]. Inaccuracies at these stages may result in complications such as root perforation, drill deviation, and failure to locate the canal [17, 19, 24]. Understanding these shortcomings is critical for refining the technique and optimizing clinical outcomes. This case report documents repeated drill deviation during static‐guided endodontics, potentially associated with drill–sleeve incompatibility and progressive wear of the resin template surrounding the sleeve, followed by successful canal localization using a microscope and ultrasonic tips. These findings highlight technical factors that should be considered during guide design and clinical execution in severe PCO cases.

## 2. Case Presentation

The present case report was written in accordance with CARE guidelines [26] and with the Declaration of Helsinki. Ethical approval was obtained from the Institutional Ethics Committee of the School of Dentistry, University of São Paulo (CAAE: 88453625.9.0000.0075, Protocol Number: 7.615.038).

A 69‐year‐old female patient with a medical history of osteoporosis (under treatment) was referred to a School of Dentistry for endodontic evaluation of the maxillary right lateral incisor. The patient reported a history of dental trauma involving this tooth approximately 1 year before presentation. According to the patient, no dental treatment was performed at the time of the traumatic event, and further information regarding the type or circumstances of the traumatic injury was unavailable. The tooth remained untreated until the onset of persistent mastication pain, when conventional endodontic access was attempted by a previous endodontist; however, the root canal could not be located. Written informed consent was obtained prior to treatment.

### 2.1. Clinical and Radiographic Findings

Clinical examination revealed no periodontal abnormalities. The tooth was nonresponsive to cold testing, with normal responses to horizontal percussion and palpation, and a positive response to vertical percussion. Given the severe PCO, the negative response to cold testing was considered inconclusive for determining pulpal status, as extensive hard‐tissue deposition may interfere with sensibility testing. Periapical radiographs indicated severe PCO with no discernible canal lumen (Figure 1A). High‐resolution CBCT (0.125‐mm voxel size, 14‐bit grayscale, 17.9 s of x‐ray exposure, 100.0 kV, and 8.0 mA—Prexion 3D Elite, Terarecon, San Mateo, United States) confirmed PCO, a widened periodontal ligament space, and evidence of prior unsuccessful access (Figure 1B–D). Based on the clinical and imaging findings, an apical diagnosis of symptomatic apical periodontitis was established. Although pulp necrosis was suspected, a definitive pulpal diagnosis could not be established based on sensibility testing alone.

**Figure 1:**
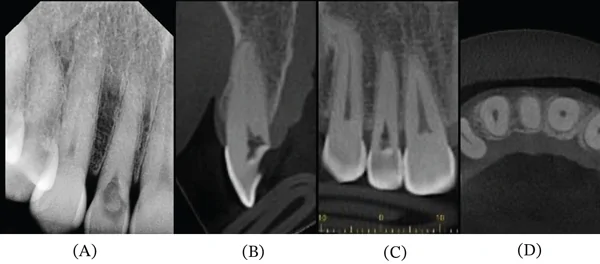
Preoperative periapical radiograph and cone‐beam computed tomography (CBCT). (A) Initial periapical radiograph. (B) Sagittal CBCT slice. (C) Coronal CBCT slice. (D) Axial CBCT slice.

### 2.2. Guided Endodontic Access: Planning and Execution

An intraoral scan was conducted using a Trios (3Shape, Copenhagen, Denmark). The generated STL file was aligned and superimposed with the CBCT images using the 3Shape Dental System software (3Shape, Copenhagen, Denmark).

A virtual guide was designed for endodontic access to the middle‐apical third of the root canal (Figure 2A). Although the total length of the tooth was 23 mm, the root canal lumen was visible only 8 mm from the apex. A virtual drill compatible with the double straight point (DSP) drill was incorporated into the software, measuring 20 mm in length and 1.0 mm in diameter, and was virtually positioned to access the root canal system (Figure 2B–D). A metallic sleeve with a height of 5 mm and an external diameter of 3 mm, compatible with the 1.0‐mm DSP drill was incorporated into the endodontic guide to improve the stability of drill positioning and better control its penetration depth. Subsequently, the design of a 3D template was sent to a 3D printer, and the endodontic guide was fabricated using S.I.N Guide 3.0 resin (S.I.N Implant System, São Paulo, Brazil) (Figure 2E). All fabrication steps were reviewed and confirmed by the endodontist.

**Figure 2:**
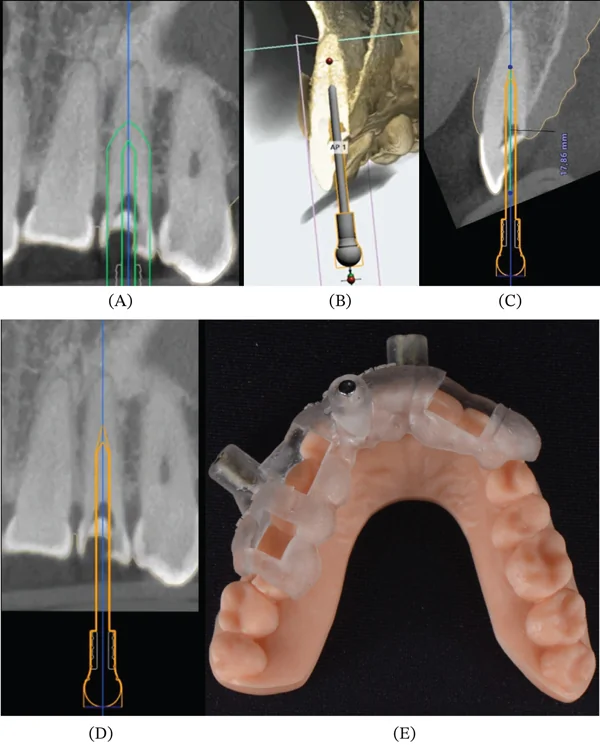
Virtual planning of the endodontic guide. (A) Guided access was repeated using the second guide; palatal drill deviation persisted. (B) Access planning with the DSP virtual drill superimposed on the 3D model. (C) Access planning with the DSP virtual drill superimposed on sagittal CBCT slice. (D) Access planning with the DSP virtual drill superimposed on coronal CBCT slice. (E) Printed model and static endodontic guide.

Under local anesthesia (2% lidocaine with 1:100,000 epinephrine—Alphacaine, DFL Indústria e Comércio S/A, Rio de Janeiro, Brazil), the provisional glass ionomer restoration and underlying cotton pellet from the previous access attempt, which was limited to the cervical third, were removed using a high‐speed round diamond drill (1014; Komet, Rock Hill, SC). The pre‐existing access cavity was left unfilled during the guided procedure. The adaptation and stability of the guide were confirmed intraorally and through the inspection windows. Stabilizing pins were deemed unnecessary, as the guide adequately covered the labial and palatal surfaces of the adjacent teeth. The 1.0‐mm DSP drill (DSP Biomedical, Campo Largo, Paraná, Brazil) attached to a VDW Silver endodontic motor (VDW, Munich, Germany—800 rpm, 4 N.cm torque) was used with saline irrigation. Despite radiographic monitoring (Figure 3A–C), upon reaching the estimated depth, a C‐Pilot #10 file (VDW, Munich, Germany) was introduced; however, canal localization was not achieved, even after repeated attempts (Figure 3D).

**Figure 3:**
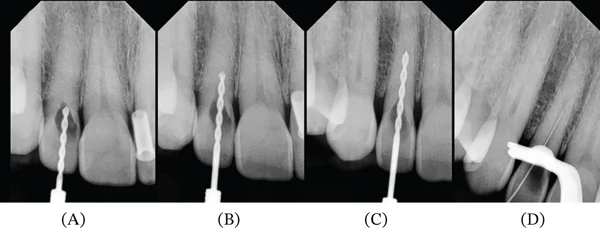
Intraoperative periapical radiographs. (A) DSP drill at the beginning of the cervical third. (B) DSP drill in the middle third. (C) DSP drill at the estimated maximum depth. (D) C‐Pilot #10 hand file attempting to locate the root canal.

Postoperative CBCT revealed palatal deviation (Figure 4A,B). The deviated access pathway created during the first guided attempt was not sealed before the second guided procedure. For the second attempt, a new CBCT scan and intraoral scan were obtained, and a new guide was planned considering the deviation created during the first guided procedure. The same DSP drill and the same type and dimensions of metallic sleeve were maintained. A second guide was fabricated, but a similar deviation occurred, with wear observed in the surrounding resin template adjacent to the metallic sleeve on the palatal aspect (Figure 4C,D).

**Figure 4:**
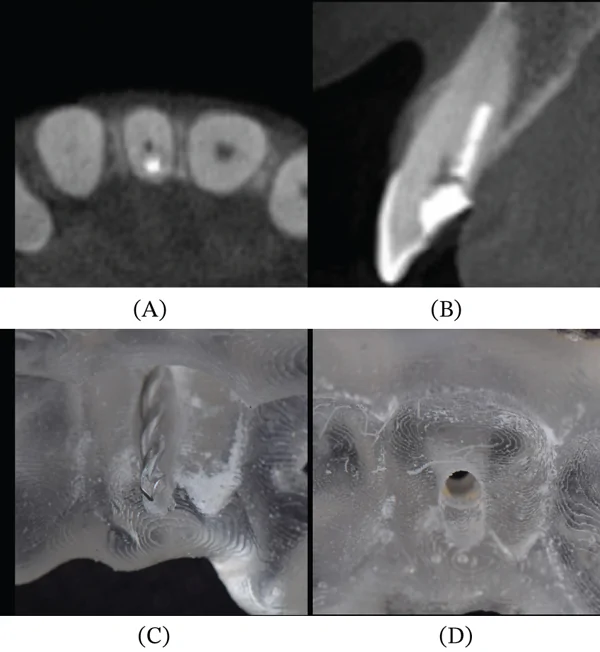
Intraoperative cone‐beam computed tomography showing deviation toward the palatal aspect and the endodontic guide after drilling. (A) Axial CBCT slice. (B) Sagittal CBCT slice. (C) Metallic sleeve and wear of the surrounding resin template on the palatal aspect. (D) Palatal resin wear adjacent to the metallic sleeve after drilling. (D′) Higher‐magnification view of the metallic sleeve ( ^∗^) shown in Figure D.

The guide inspection revealed that the metallic sleeve measured 5 mm in height and the active portion of the DSP drill, 12 mm (Figure 5A,B). No visible wear of the metallic sleeve was observed. Instead, wear occurred in the surrounding resin template on the palatal aspect. The 5‐mm metallic sleeve was shorter than the 12‐mm active cutting portion of the DSP drill, allowing the active portion of the drill to extend beyond the sleeve and contact the surrounding resin template. The observed resin wear and the discrepancy between sleeve height and the active cutting length of the drill suggest that inadequate guidance of the active cutting portion may have contributed to progressive enlargement of the guide pathway and subsequent drill deviation (Figure 5D,E).

**Figure 5:**
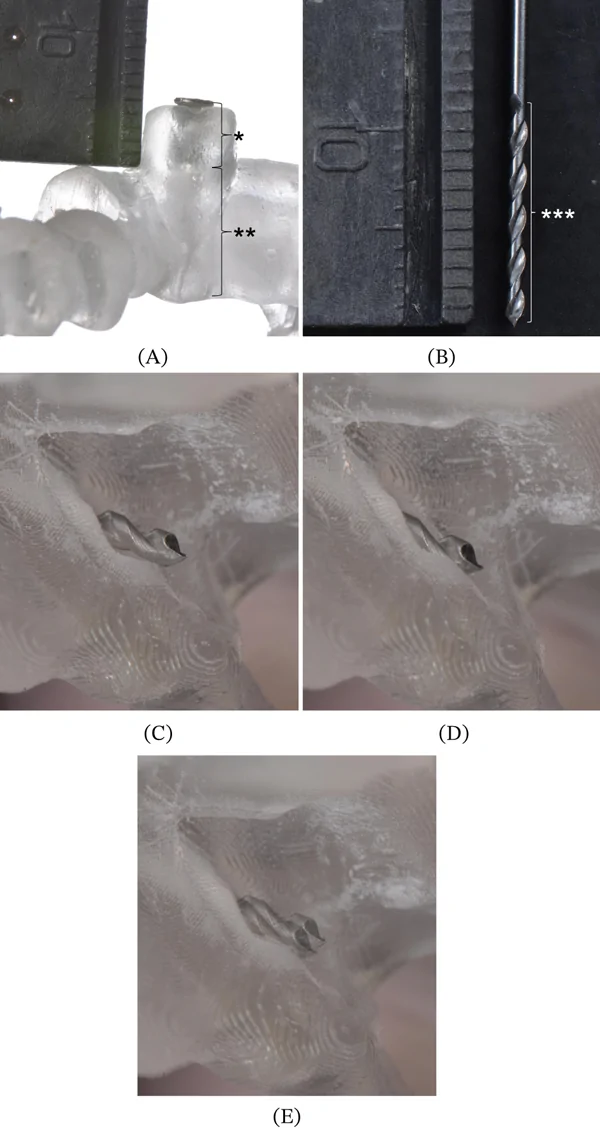
Guide inspection and drill–sleeve relationship. (A) Metallic sleeve measuring 5 mm in height ( ^∗^) and the area of drill contact with the surrounding resin template ( ^∗∗^). (B) Active portion of the DSP drill ( ^∗∗∗^) measuring 12 mm in length. (C) Buccal movement of the drill. (D) Palatal movement of the drill. (E) Superimposition of Panels C and D illustrating the range and direction of lateral drill displacement.

### 2.3. Conventional Management and Outcome

After discussion with the patient, the canal was successfully localized under surgical microscopy (3x–16x magnification) (DF Vasconcelos, Valença, Rio de Janeiro, Brazil), using Finder ultrasonic tip (Helse Ultrasonics, Santa Rosa de Viterbo, Sao Paulo, Brazil), and a #10 K‐file (Dentsply Maillefer). Upon canal access, necrotic tissue remnants were observed, with no bleeding or other evidence of vital pulp tissue. These intraoperative findings supported the initial clinical suspicion of pulp necrosis. The working length (21 mm) was confirmed using an apex locator (Romi Apex A15; Romidan, Sao Paulo, Brazil) and radiographic examination (Figure 6A).

**Figure 6:**
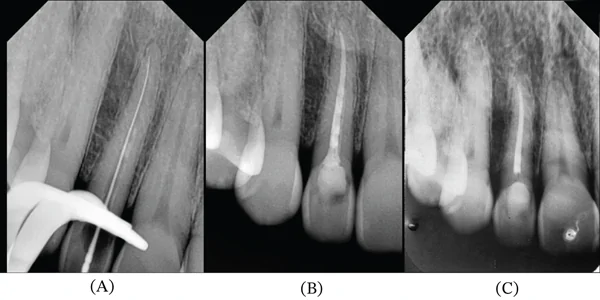
Intraoperative, posttreatment, and follow‐up periapical radiographs. (A) Intraoperative periapical radiograph showing canal localization and a file positioned at the working length (21 mm). (B) Periapical radiograph after root canal obturation and temporary restoration. (C) Three‐year follow‐up periapical radiograph showing the definitive composite restoration and no radiographic signs of apical pathosis.

The root canal was prepared to a final apical size of 35/0.05 (Logic, Easy Equipamentos Odontologicos Brazil), with copious irrigation using 40 mL of 2.5% sodium hypochlorite [27]. Calcium hydroxide intracanal dressing (Ultracal XS, Ultradent, United States) was placed for 21 days [28]. After this period, each irrigant was ultrasonically activated in three 30‐s cycles (Irrisonic 01; Helse, Santa Rosa do Viterbo, Sao Paulo, Brazil): 5 mL of 2.5% sodium hypochlorite and 5 mL of 17% EDTA. The root canal filling was performed using Bio‐C Sealer (Angelus, Brazil) and warm vertical condensation technique. The palatal defect was restored with a self‐etching dual‐cure resin cement (AllCem Core, FGM Produtos Odontológicos, Joinville, Santa Catarina, Brazil). A dual‐cure material was selected because the depth and configuration of the defect could limit light penetration, whereas the chemical‐curing component allowed polymerization in deeper areas. The coronal access was subsequently sealed with glass ionomer cement (3 M Oral Care, St Paul, Minnesota) as an interim restoration (Figure 6B). Considering the prolonged duration of the clinical procedure and the patient’s upcoming travel, definitive restorative treatment was postponed. One month later, the patient remained asymptomatic, and the definitive composite resin restoration was performed in the coronal access (Filtek Z350 XT, 3 M Oral Care, St Paul, Minnesota). A 3‐year clinical and radiographic follow‐up showed a satisfactory outcome (Figure 6C). The clinical timeline is summarized in Table 1.

**Table 1:** Clinical timeline of the case.

Time point	Clinical event
Approximately 1 year before presentation	Dental trauma involving the maxillary right lateral incisor; no treatment was performed.
Before referral	Onset of pain during mastication, followed by an unsuccessful conventional attempt to locate the obliterated root canal.
Initial assessment	Severe PCO identified and symptomatic apical periodontitis diagnosed.
Treatment planning	CBCT and intraoral scanning followed by virtual planning and fabrication of a static endodontic guide.
First guided attempt	Guided access performed; deviation from the planned trajectory identified.
Second planning	A new guided access was planned and a second static endodontic guide was fabricated.
Second guided attempt	Guided access was repeated using the second guide; palatal drill deviation persisted.
Conventional treatment	Canal successfully located under magnification and ultrasonic assistance; root canal prepared and intracanal medication placed.
21 days later	Root canal obturation with gutta‐percha and Bio‐C Sealer was completed. The deviated access pathway was sealed with dual‐cure resin cement. Temporary coronal sealing was performed with glass ionomer cement.
1 month later	The patient remained asymptomatic, and definitive restoration with composite resin was performed.
3‐year follow‐up	The tooth remained asymptomatic, with satisfactory clinical and radiographic findings.

### 2.4. Patient Perspective

From the patient′s perspective, severe pain during mastication persisted throughout the guided treatment attempts. Following successful canal localization using the conventional approach and root canal instrumentation, the pain resolved completely, and the patient remained asymptomatic and satisfied with the treatment outcome during follow‐up.

## 3. Discussion

Most of the literature on guided endodontics consists of successful clinical case reports, primarily focused on locating severely obliterated root canals [6, 8, 11, 13, 17, 29]. Although reporting successful outcomes provides valuable insight into the benefits of guided endodontics, openly discussing procedural failures is equally critical. However, complications and procedural failures have been less frequently reported [12, 19, 24], limiting a comprehensive understanding of the potential risks and limitations of static guided endodontics in clinical practice. The present case highlights technical limitations of static‐guided endodontics that should be considered during planning and clinical execution.

Tissue preservation is an important factor directly associated with the long‐term prognosis of endodontically treated teeth. In this context, guided endodontics aligns with the principles of minimally invasive endodontics by enabling conservative access preparation and reducing unnecessary dentin removal [29]. Similarly, the use of instruments with smaller taper contributes to maintaining radicular dentin thickness and preserving tooth structural integrity, potentially reducing the risk of root fracture over time [30]. Therefore, although guided approaches aim to minimize substance loss, technical inaccuracies may compromise these advantages and reinforce the importance of careful planning and execution.

The CBCT scanner′s accuracy and the image′s spatial resolution can directly affect the precision of guided drilling procedures. In this case report, CBCT acquisition adhered to established guidelines [12, 31, 32], ensuring high‐resolution visualization of the obliterated canal. However, its accuracy alone cannot overcome technical pitfalls, such as drill‐sleeve mismatches.

An important consideration is that the planning, design, and drills used in endodontic guides are often adapted from tools originally developed for other purposes, particularly implantology. This adaptation may impose certain limitations, especially when managing teeth with narrow or severely obliterated root canals [20]. Moreover, there is still no consensus regarding which instrument is the most effective or safest overall, highlighting the need for further comparative studies to establish standardized protocols.

Notably, drill diameter has been shown to influence both accuracy and dentin removal; whereas thinner drills are more conservative, they also tend to offer less stability during the procedure [33]. Reported deviations in the literature range from 1.81° to 2.8° in angular deviation, with coronal deviations between 0.13 and 0.21 mm, and apical deviations from 0.17 to 0.47 mm [23, 34]. Such deviations may lead to critical complications, including root perforations [19, 35], particularly in canals with more apically extended obliteration, in which the risk of drill deviation has been reported to increase by up to 15‐fold [14]. Furthermore, failures are more likely to occur in anatomically challenging teeth, such as maxillary lateral incisors with thin and curved roots, where precise navigation is especially demanding [18].

Several technical factors can contribute to such outcomes, including the template type, sleeve height, drilling distance, and drill–sleeve mismatch [19, 25]. Although DSP drills offer enhanced control, reduced dentin wear, and lower thrust force, their optimal performance depends heavily on precise alignment with the sleeve [7]. Sleeve length also plays a critical role. Longer sleeves improve stability and reduce the risk of deviation, particularly in the apical region [6, 25]. In contrast, shorter sleeves are associated with greater intrinsic angular error, with deviations increasing as the sleeve length decreases [25, 34]. A minimum sleeve length of 8 mm is therefore recommended to ensure adequate stability [24]. This observation is consistent with findings from implantology, where open‐sleeve designs have been associated with reduced accuracy in posterior regions compared with closed‐sleeve templates [7, 36].

In the present case report, the combination of a relatively short sleeve and a disproportionately long active portion of the DSP drill, combined with potential wear of the resin guide during drilling, may have permitted increased lateral movement once the drill extended beyond the sleeve, thereby contributing to the observed deviations. These findings highlight the critical need for meticulous planning, particularly to ensure drill‐sleeve compatibility and to optimize guide design. Although the short sleeve was selected to achieve the required drilling depth, a sequential approach using a longer sleeve initially and a shorter sleeve for deeper access might have provided greater drill stability. In this context, alternative guide designs, such as sleeveless systems, as well as drills with more restricted active cutting areas, may potentially reduce unintended contact with the guide and minimize resin wear during drilling procedures. The limited literature on these specific technical variables underscores the need for standardized protocols to minimize risks and improve clinical outcomes.

Although the observed palatal resin wear and the recurrence of deviation in the same direction support a mechanical contribution related to the drill–sleeve–guide relationship, this mechanism cannot be established as the sole cause of failure. Other factors inherent to the static‐guided workflow may also have contributed, including incomplete guide seating, errors during CBCT–surface scan superimposition, manufacturing and printing tolerances, drill–sleeve clearance, the presence of a pre‐existing access cavity, operator‐applied pressure, and the specific anatomy of the maxillary lateral incisor. Therefore, the observed deviation was likely multifactorial, although the pattern of guide wear provided clinical evidence supporting a mechanical component.

Accordingly, future studies should prioritize the development and validation of longer guide sleeves and endodontic‐specific drills with active cutting lengths precisely matched to the sleeve dimensions. Such advancements may help reduce procedural deviations and enhance the precision and reliability of guided endodontics, particularly in cases involving severely obliterated canals. It is also worth noting that systematic reviews highlight a concerning lack of failure reporting [25]; only two perforations have been documented across 45 studies, suggesting a potential publication bias.

Additionally, a potential cause of deviations in guided endodontics, particularly in teeth with previously attempted conventional or guided access [14], is the drill′s natural tendency to follow paths of least resistance. Considering that an intentional clearance of approximately 0.2 mm is typically planned between the drill and the metallic sleeve to ensure frictionless rotation, any pre‐existing access cavity, even if minimal, creates a lower resistance area. Consequently, the drill may preferentially deviate into these existing paths, increasing the likelihood of unintended resin wear on the guide sleeve and subsequent procedural deviations. Therefore, an advisable clinical step prior to guided endodontic procedures in previously accessed teeth may be to seal any existing conventional or original access cavities with composite resin. This preventive measure would ensure consistent resistance across the surface, thereby encouraging the drill to establish a new and accurate pathway according to the planned guided access trajectory. However, this interpretation has not been experimentally validated. Therefore, sealing previous access cavities should not be regarded as an established protocol, and further studies are needed to determine whether this strategy can effectively reduce drill deviation.

Although guided endodontics provides valuable assistance, clinical expertise remains indispensable for successful outcomes [3, 4, 37]. The ability to recognize deviations, adapt intraoperatively, and rely on conventional techniques—such as magnification and ultrasonic tips—was pivotal in achieving a favorable result. Thus, guided endodontics should be considered an adjunct to, rather than a replacement for, expert clinical judgment and technical skill. These challenges highlight the need for further research and the development of endodontic‐specific drills and sleeves to enhance procedural accuracy and reduce technical errors in complex PCO cases.

This report adds to the current literature by documenting a technical failure of static guided endodontics potentially associated with drill–sleeve misalignment and guide wear during the management of a severe PCO. Unlike most published studies that focus primarily on successful outcomes, this case highlights a clinically relevant limitation of guided endodontics and reinforces the importance of meticulous guide validation and operator proficiency in conventional techniques when guided access fails.

One limitation of the static‐guided approach in the present case was the inability to monitor and adjust the drilling trajectory in real time. Dynamic navigation may represent an alternative in such cases by allowing intraoperative trajectory adjustments; however, its implementation requires specific equipment and operator training [38]. Additional inherent limitations of static‐guided endodontics include the space required for guide and drill insertion in patients with limited mouth opening, template guided, heat generation, inability to follow curved trajectories, additional radiation exposure associated with CBCT, increased cost and planning time, and limited intraoperative adaptability [24, 25].

Based on the present case, careful evaluation of drill‐sleeve compatibility appears essential to minimize mechanical instability and guide wear during guided endodontic procedures. In addition, excessive friction between the rotating drill and the guide walls should be avoided, as this may contribute to progressive resin wear and compromise drilling accuracy. Sealing a previous access cavity before a new guided attempt may be considered as a potential strategy to provide a more uniform drilling surface; however, this hypothesis requires experimental validation.

This report has several limitations. First, it describes a single clinical case, and the observations therefore cannot be generalized to all static‐guided endodontic procedures. Although the pattern of palatal guide wear and repeated deviation in the same direction support the proposed mechanical explanation, the mechanism responsible for the failure was not experimentally confirmed. In addition, the extent of guide wear and drill deviation was not quantitatively measured. Finally, the findings are specific to the drill, sleeve, guide material, manufacturing workflow, printer, and tooth anatomy used in this case and should not be extrapolated to other guided systems, materials, manufacturing technologies, or tooth types.

In conclusion, static‐guided endodontic access may be affected by procedural deviations despite careful planning. Clinicians should recognize the technical limitations of guided endodontics—particularly regarding sleeve design, drill selection, and drill‐sleeve alignment—and verify drill–sleeve compatibility before clinical use. In the present case, the relationship between the short metallic sleeve, the longer active cutting portion of the drill, and the observed guide wear may have contributed to the repeated palatal deviation; however, other technical and anatomical factors cannot be excluded. In this case, microscope‐assisted access and ultrasonic tips enabled successful canal localization after guided access failed. These observations reinforce the importance of clinician experience, knowledge of root canal anatomy, and proficiency in conventional techniques when managing procedural complications associated with guided endodontics.

## Author Contributions

Stephanie Isabel Diaz Zamalloa: conceptualization, data curation, formal analysis, investigation, methodology, software, validation, writing—original draft, and writing—review and editing. Iandara Lima Scardini: investigation, software, validation, writing—original draft, and writing—review and editing. Shirley Maklane Gomes Soares: data curation, formal analysis, investigation, software, and writing—review and editing. Daniel de Almeida Decurcio: investigation, methodology, validation, and writing—review and editing. Marcelo dos Santos: conceptualization, data curation, formal analysis, investigation, methodology, validation, and writing—review and editing. Celso Luiz Caldeira: investigation, methodology, validation, and writing—review and editing.

## Funding

No funding was received for this manuscript.

## Disclosure

All authors have read and approved the final version of the manuscript. Iandara Lima Scardini had full access to all of the data in this study and takes complete responsibility for the integrity of the data and the accuracy of the data analysis.

## Ethics Statement

This case report was written in accordance with CARE guidelines and with the Declaration of Helsinki. Ethical approval was obtained from the Institutional Ethics Committee of the School of Dentistry, University of São Paulo (CAAE: 88453625.9.0000.0075, Protocol number: 7.615.038).

## Consent

A written informed consent was obtained from the patient. No data on the paper reveals the patient′s identity.

## Conflicts of Interest

The authors declare no conflicts of interest.

## Data Availability

Data sharing is not applicable to this article as no datasets were generated or analyzed during the current study.
